# ^18^F-Fluorothymidine PET-CT for Resected Malignant Gliomas before Radiotherapy: Tumor Extent according to Proliferative Activity Compared with MRI

**DOI:** 10.1371/journal.pone.0118769

**Published:** 2015-03-04

**Authors:** Fen Zhao, Minghuan Li, Zhiheng Wang, Zheng Fu, Yunfeng Cui, Zhaoqiu Chen, Jinming Yu

**Affiliations:** 1 Department of Radiation Oncology, Shandong Cancer Hospital, Shandong Academy of Medical Sciences, Jinan, China; 2 Key Laboratory of Radiation Oncology of Shandong Province, Shandong Cancer Hospital and Institute, Jinan, Shandong, China; 3 Department of Radiation Oncology, Duke University Medical Center, Durham, NC, United States of America; 4 Department of Nuclear Medicine, Shandong Cancer Hospital and Institute, Jinan, Shandong, China; 5 Department of radiology, Shandong Cancer Hospital and Institute, Jinan, Shandong, China; University of Alabama at Birmingham, UNITED STATES

## Abstract

**Objective:**

To compare the presence of post-operative residual disease by magnetic resonance imaging (MRI) and [18F]fluorothymidine (FLT)-positron emission tomography (PET)-computer tomography (CT) in patients with malignant glioma and to estimate the impact of ^18^F-FLT PET on the delineation of post-operative target volumes for radiotherapy (RT) planning.

**Methods:**

Nineteen patients with post-operative residual malignant gliomas were enrolled in this study. For each patient, ^18^F- FLT PET-CT and MRI were acquired in the same week, within 4 weeks after surgery but before the initiation of RT. The PET-CT and MRI data were co-registered based on mutual information. The residual tumor volume defined on the ^18^F-FLT PET (Vol-PET) was compared with that of gadolinium [Gd] enhancement on T1-weighted MRI (Vol-T1) and areas of hyperintensity on T2-weighted MRI (Vol-T2).

**Results:**

The mean Vol-PET (14.61 cm^3^) and Vol-T1 (13.60 cm^3^) were comparable and smaller than the mean Vol-T2 (32.93 cm^3^). The regions of ^18^F-FLT uptake exceeded the contrast enhancement and the hyperintense area on the MRI in 14 (73.68%) and 8 patients (42.11%), respectively. In 5 (26.32%) of the 19 patients, Vol-PET extended beyond 25 mm from the margin of Vol-T1; in 2 (10.53%) patients, Vol-PET extended 20 mm from the margin of Vol-T2. Vol-PET was detected up to 35 mm away from the edge of Vol-T1 and 24 mm away from the edge of Vol-T2. In 16 (84.21%) of the 19 patients, the Vol-T1 extended beyond the Vol-PET. In all of the patients, at least some of the Vol-T2 was located outside of the Vol-PET.

**Conclusions:**

The volumes of post-operative residual tumor in patients with malignant glioma defined by ^18^F-FLT uptake on PET are not always consistent with the abnormalities shown on post-operative MRI. Incorporation of ^18^F-FLT-PET in tumor delineation may have the potential to improve the definition of target volume in post-operative radiotherapy.

## Introduction

Malignant gliomas are the most common primary central nervous system tumors in adults. Maximal surgical resection followed by external-beam radiotherapy (EBRT) and adjuvant temozolomide (TMZ) chemotherapy is the standard treatment to date. The prognosis of patients with malignant glioma remains poor [1]. The most common failure pattern is the progression of the residual lesion or recurrence of the tumor bed, which is the so-called in-field failure [2–4]. Precise residual tumor identification and target volume delineation are of crucial importance for radiotherapy after surgery. However, magnetic resonance (MR) technology, such as magnetic resonance spectroscopy (MRS), diffusion-weighted imaging (DWI), perfusion-weighted imaging (PWI), and cerebral blood volume measurements with dynamic susceptibility-weighted contrast material-enhanced (DSC) MR imaging (MRI), were reported to be extremely useful in the differential diagnosis, grading, and treatment response evaluation of gliomas [5–9]. Some of these evolving technologies have not been comprehensively applied clinically. Conventional MRI and computed tomography (CT) are still considered the standard methods for post-surgical target volume delineation. However, in patients treated with neurosurgery, the persistent tumor is sub-optimally differentiated from the nonspecific post-operative changes in either conventional CT or MRI because of treatment-related blood—brain barrier (BBB) disturbances. Additionally, these post-operative changes can persist for months after surgery [10–12]. In this context, it is clearly justified to search for new methods to define the tumor volume more precisely in post-operative imaging.

In recent years, positron emission tomography (PET) has been introduced in the management of primary brain tumors. PET imaging can reveal specific biological events and has the potential to complement the anatomical information derived from traditional radiological techniques. 3′- Deoxy-3′-[18F]-fluorothymidine (^18^F-FLT), an analog of thymidine, has emerged as a promising PET tracer for evaluating tumor proliferation in various malignant brain tumors [13–15]. Because of the low uptake of ^18^F-FLT in intact brain tissue, ^18^F-FLT PET provides a low-background cerebral image; thus, it is considered an attractive imaging method for malignant brain tumors. Earlier publications have shown that ^18^F-FLT PET is useful in the noninvasive grading, prognostic assessment, discrimination of recurrent tumors from post-treatment radio-necrosis, and early outcome predictions of systemic therapy in patients with malignant glioma [16, 17]. Additionally, Jacobs *et al*. reported that ^18^F-FLT-, L-(methyl-11C)-labeled methionine (^11^C-MET)-PET, as well as gadolinium (Gd)-enhanced MRI yield complementary information concerning the activity and extent of gliomas [18]. Several studies have reported the impact of biological imaging, such as MRS [19], ^11^C-MET-PET [20, 21], and O-(2-(F-18)fluoroethyl)-L-tyrosine-(^18^F-FET)-PET[22], on the definition of target volume for radiation therapy in glioma patients. However, to our knowledge, little data in the literature have quantified tumor extension in ^18^F-FLT PET and MRI and have compared these two imaging modalities using image fusion.

The purpose of this prospective study was to compare the post-operative volumes of residual disease defined by ^18^F-FLT PET and MRI in patients with malignant gliomas prior to receiving radiotherapy (RT). Furthermore, we also aimed to estimate the potential impact of ^18^F-FLT PET on the delineation of post-operative target volumes for RT planning.

## Patients and Methods

### Patients

From May 2012 to August 2013, 19 adult patients (13 males and 6 females; median age, 52 years) with histologically confirmed malignant astrocytoma were included in the present study. Surgical resection was performed in all of the cases. The inclusion criteria were the diagnosis (or suspicion) of residual tumor on MRI performed within 24 hours after surgery and/or the intra-operative diagnosis of possible residual tumor. Twelve of the 19 patients had World Health Organization (WHO) grade IV glioblastomas (GBM), and the other 7 patients had WHO grade III anaplastic astrocytomas. The Karnofsky performance status (KPS) scores of all patients were between 70 and 100. For each patient, MRI and ^18^F- FLT PET-CT were performed during the same week, within 4 weeks after surgery but before the initiation of radiotherapy (RT). The steroid dose was not changed during this week of MRI and PET examinations. The present prospective study was approved by the Institutional Review Board of Shandong Cancer Hospital and Institute, and all patients provided written informed consent. The characteristics of the included patients are listed in Table 1 and S1 Table.

**Table 1:** Patient’s characteristic (n = 19).

	N (%)
Age, median(range)	52(26–67)
Gender(n)	
Male(n)	13 (68.42)
Female(n)	6 (31.58)
KPS	
90–100	5 (26.32)
80–90	10 (52.63)
70–80	4 (21.05)
Anaplastic astrocytoma	7 (36.84)
Glioblastomas	12 (63.16)
WHO grade	
Ⅲ	7 (36.84)
Ⅳ	12 (63.16)
Extent of Surgery	
Subtotal resection	10 (52.63)
Partial resection	9 (47.37)

### MRI

MRI was performed using a Philips 3.0 Tesla scanner Achieva 3.0T X-series (Philips Medical Systems, Best, The Netherlands). The patient’s head was immobilized by individual thermoplastic mask fixation during the MRI examination. Because the standard head coil was too small to use with thermoplastic mask fixation, acquisition was performed using a standard body coil. T1-weighted imaging was performed using a fast field echo sequence with a repetition time (TR) of 433 ms, an echo time (TE) of 2.3 ms, a flip angle (FA) of 70°, and a bandwidth of 173.7 Hz/pixel; T2-weighted imaging was performed using a turbo spin echo sequence with a TR of 4393 ms, a TE of 380 ms, an FA of 90°, and a bandwidth of 255.1 Hz/pixel. The field of view (FOV) was 87.5 mm. Contrast-enhanced T1-weighted imaging was acquired 180 seconds after the administration of gadolinium diethylenetriaminepentaacetic acid (DTPA) 6.6±0.9mmol (Magnevist; 0.1 mmol/kg body weight; Bayer Schering Pharma, Osaka, Japan), using the same parameters as those for T1-weighted imaging. The slice thickness was 4.25 mm with one signal average and a 512×512 acquisition matrix.

### Radiopharmaceuticals and ^18^F-FLT PET scan

^18^F-FLT was synthesized by a cyclotron (Mini Trace; GE Healthcare, Milwaukee, WI, USA) and a synthesizer (TracerLab FxFN; GE Healthcare) and was prepared according to the method described by Yue et al. [23]. The products had to meet pre-specified criteria (e.g., radiochemical yield >10%, radiochemical purity >95%) to qualify for use in imaging.

^18^F-FLT PET-CT studies were performed using an integrated PET system (Discovery LS; GE Medical Systems, Milwaukee, WI, USA), and the patients were scanned in the same position (immobilized with a head mask) as they were for the MRI scan. The imaging system enabled the simultaneous acquisition of 35 transverse PET slices per FOV, with an axial sampling thickness of 4.25 mm, for a total axial FOV of 14.5 cm.

No specific dietary instructions were given to the patients before the ^18^F-FLT PET-CT study. Patients rested for 15 minutes prior to the administration of FLT (374.2±36.7 MBq). Next, patients were instructed to rest peacefully for 53±6 minutes (used for uptake) after the injection. Noncontrast CT (parameters: 80 mA, 140 kV, 0.8 s/tube rotation, and reconstructed slice thickness of 4.25 mm) for attenuation correction was first performed covering the vertex of skull to the second cervical vertebra using a multi-detector helical CT scanner. PET was then acquired immediately in the three-dimensional mode with a scan time of 7 minutes per bed position. Data were reconstructed using the ordered subsets expectation maximization (OSEM) algorithm with two iterations and 16 subsets, applying a matrix size of 128×128. The PET images were viewed on a Xeleris workstation (GE Healthcare).

### PET-CT and MRI-CT image fusion

All of the images were transferred to the Pinnacle3 treatment planning system (Philips Radiation Oncology Systems, Milpitas, CA, USA) for analysis. The Syntegra toolkit in the Pinnacle3 TPS was used to perform rigid body image registration automatically. The T1- and T2-weighted MR images were aligned automatically with the CT images of PET-CT, respectively, using the normalized mutual information metric. Manual correction was then performed, if necessary, by the investigator and a senior staff member to mitigate the mismatching based on anatomies (such as clivus, orbital cavity, nasal cavity, lateral ventricles, mastoid air cells, and optic nerve) [24]. When contoured on the MR or PET images, the contours could be simply transferred and superimposed onto the CT image based on the fusion.

### Image analysis

The images were analyzed visually by two experienced nuclear medicine radiologists independently. The maximum standardized uptake value (SUV_max_) of the tumor was derived by placing a circular region-of-interest (ROI) with a diameter of 10 mm on the tumor region with the highest tracer uptake. The standardized uptake value (SUV) of the reference tissue was calculated as the mean SUV inside a commensurate ROI placed manually on the uninvolved contralateral hemisphere by referring to diagnostic MRI and MRS. If it was impossible to position the reference ROI at the contralateral hemisphere due to the tumor location, the ROI was placed on intact brain regions in the plane that showed the maximum ^18^F-FLT uptake. The tumor-to-normal brain tissue (T/N) ratio was determined by dividing the SUV_max_ of the tumor by the SUV_mean_ of the reference brain tissue.

### Biological and morphological tumor volume delineation

Each image data set was contoured by three investigators, and the final determination of tumor delineation was obtained by consensus among them. The residual tumor volume after surgery defined by the ^18^F-FLT uptake was delineated semi-automatically in the ^18^F-FLT PET images (Vol-PET). For the target margin in all of the patients, we used a threshold value of 2.0 for the T/N ratio (18). The high uptake regions in the normal tissues, such as the lacrimal glands or mucosa, were subtracted manually from the Vol-PET. The contrast-enhancement lesions on T1-weighted MRI after subtraction of the distinct areas of hemorrhage (defined on T1-native images) were contoured as Vol-T1. The hyperintense areas on the T2-weighted images, including residual tumor tissue, edema, post-operative changes, and the resection cavity, were delineated as Vol-T2. The volumes of ^18^F-FLT uptake hyperactivity and the abnormal signal changes on MRI were calculated by the treatment planning system.

We also assessed the following parameters: 1) summed Vol: for those with ^18^F-FLT uptake beyond the abnormity signal changes on MRI, or vice versa, the summed volume MRI/PET (summed Vol-T1/PET and summed Vol-T2/PET) were calculated; summed Vol-T1/PET = Vol-T1∪Vol-PET and summed Vol-T2/PET = Vol-T2∪Vol-PET, where ∪ indicates the sum; 2) intersection Vol: intersection Vol-T1/PET = Vol-T1∩Vol-PET and intersection Vol-T2/PET = Vol-T2∩Vol-PET, where ∩ indicates the intersection; 3) the portion of Vol-PET that is not overlapping with Vol-T1 (or Vol-T2) was defined as Vol-(PET minus T1) (or Vol-(PET minus T2)). The portion of Vol-T1 (or Vol-T2) that is not overlapping with Vol-PET was defined as Vol-(T1 minus PET) (or Vol-(T2 minus PET)). The maximum distance between the margin of FLT uptake and that of the MRI changes for each patient was quantified (in mm) in the CT image, which was fused with PET and MRI, respectively.

## Results

**Fig 1:**
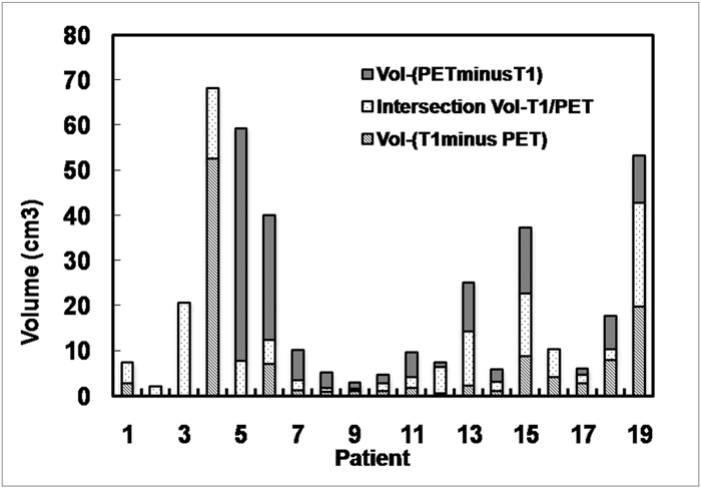
Comparison of tumor volumes defined by T1-weighted MRI with contrast enhancement and ^18^F-FLT PET. For all 19 cases, the mean Vol-T2 was 32.93 cm^3^ (range, 3.3–106.60 cm^3^). The average summed volume Vol-(T2∪PET) was 36.36 cm^3^, and the average intersection Vol-(T2 ∩ PET) was 11.12 cm^3^. The average Vol-(T2 minus PET) was 21.78 cm^3^ (range, 0.8–83.20 cm^3^), and the average Vol-(PET minus T2) was 3.43 cm^3^ (range, 0–17.5 cm^3^).

**Fig 2:**
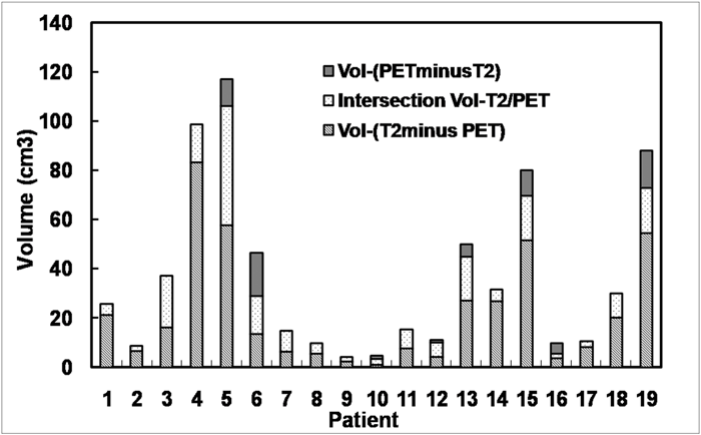
Comparison of tumor volumes defined by T2-weighted MRI and ^18^F-FLT PET. Based on the PET/CT and MRI/CT fusion images, the tumor contours derived from PET and MRI images were superimposed together on CT images and compared. In two patients (10.5%), the regions elevated between ^18^F-FLT uptake in PET images and the Gd enhancement in T1-weighted MR images corresponded exactly to each other. In 14 patients (73.68%), the ^18^F-FLT uptake was detected beyond the scope of Gd enhancement up to 35 mm from the tumor margin in MRI (range, 2–35 mm). In 5 (26.3%) of the 19 patients, ^18^F-FLT uptake extended beyond 25 mm from the margin of Gd enhancement. Additionally, in 16 patients (84.2%), the Gd enhancement was located outside the ^18^F-FLT uptake range (Table 3 and Fig. 3).

The comparisons between the tumor volumes defined by ^18^F-FLT PET and MRI are presented in Fig. 1 and Fig. 2, respectively. The statistical results are listed in Table 2 and S1 Table.

**Fig 3:**
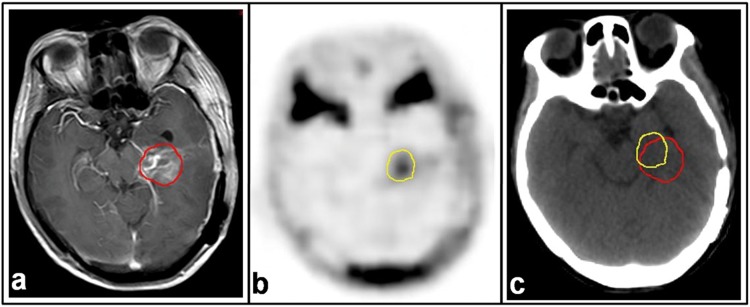
MRI and ^18^F-FLT PET images for a patient with glioblastoma (GBM). Images were taken 21 days post-operatively and 2 days before radiotherapy. a. T1-weighted MRI with contrast enhancement. b. ^18^F-FLT PET images. c. CT image of PET-CT scan. Residual tumor regions defined by T1-MRI (red line) and ^18^F-FLT PET (yellow line) are superimposed on the CT image.

**Table 2:** Results of tumor volumes measured by ^18^F-FLT PET *vs*. T1- and T2-weighted MRI (cm^3^).

Parameters	Min	Max	Mean	SD
Vol-PET	1.80	59.40	14.61	15.09
Vol-T1	1.70	78.20	13.60	18.58
Vol-T2	3.30	106.60	32.93	31.81
Intersection Vol-T1/PET	0.5	23.10	6.91	6.89
Summed Vol-T1/PET	2.20	78.2	21.24	22.16
Vol-(T1minus PET)	0.00	52.72	6.16	12.23
Vol-(PETminusT1)	0.00	51.60	7.71	12.68
Intersection Vol-T2/PET	1.80	48.40	11.12	11.18
Summed Vol-T2/PET	3.90	117.60	36.36	34.96
Vol-(T2 minus PET)	0.80	83.20	21.78	23.30
Vol-(PETminusT2)	0.00	17.50	3.43	5.69

For the entire group, the mean Vol-T1 was 13.60 cm^3^ (range, 1.7–78.20 cm^3^). The mean Vol-PET was 14.61 cm^3^ (range, 1.8–59.40 cm^3^). The average summed volume Vol-(T1∪PET) was 21.24 cm^3^, and the average summed Vol-(T1∩PET) was 6.91 cm^3^. The mean Vol-(T1 minus PET) was 6.16 cm^3^ (range, 0–52.70 cm^3^), and the mean Vol-(PET minus T1) was 7.71 cm^3^ (range, 0–51.60 cm^3^).

**Table 3:** Comparison of tumor extent defined by ^18^F-FLT uptake in PET image and abnormal signal change on MR image.

Outcomes	N (%)
^18^F-FLT uptake corresponding exactly to contrast-enhancement	2 (10.53%)
^18^F-FLT uptake beyond abnormal contrast-enhancement	14 (73.68%)
Abnormal contrast-enhancement beyond ^18^F-FLT uptake	16 (84.21%)
^18^F-FLT uptake corresponding exactly to hyperintensity areas	0 (0%)
^18^F-FLT uptake beyond hyper-intensity areas	8 (42.11%)
Hyper-intensity areas beyond ^18^F-FLT uptake	19 (100%)

**Fig 4:**
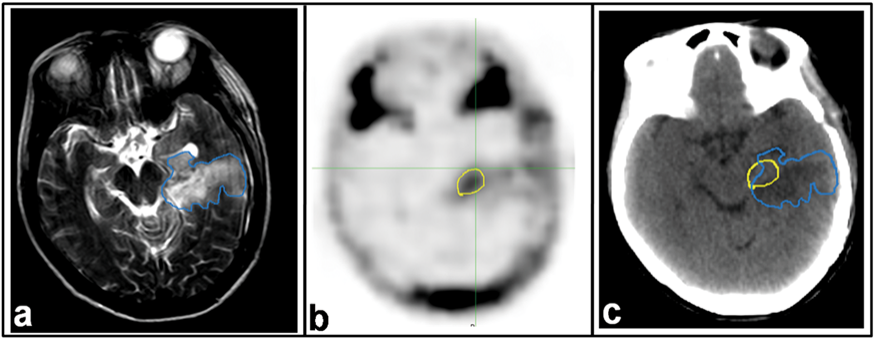
MRI and ^18^F-FLT PET-CT images from a patient with GBM. Images were taken 21 days post-operatively and 2 days before radiotherapy. a. T2-weighted MRI. b. ^18^F-FLT PET image. c. CT image of PET-CT scan. Residual tumor regions defined by T2-MRI (blue line) and ^18^F-FLT PET (yellow line) are superimposed on the CT image.

The extents of Vol-PET and Vol-T2 differed in all 19 patients. ^18^F-FLT uptake extended beyond the scope of the hyperintense area in 8 of the 19 patients (42.11%); however, in all patients (100%), the region of hyperintensity on T2-weighted MRI extended beyond the ^18^F-FLT uptake area (Table 3 and Fig. 4). ^18^F-FLT uptake was measured up to 24 mm (range, 0–24 mm) outside the margin of the hyperintense areas on T2-weighted MRI. In 2 (10.52%) of the 19 patients, ^18^F-FLT uptake extended beyond 20 mm from the margin of the hyperintense areas (Table 3 and Fig. 4).

## Discussion

In the present study, we compared the residual tumor contours defined by MRI and ^18^F-FLT PET-CT in post-operative patients with malignant gliomas. First, we found that the regions of high ^18^F-FLT uptake on PET are not consistent with those of contrast enhancement on T1-weighted MRI and hyperintense areas on T2-weighted MRI. These findings are consistent with those of previous reports that compared the residual tumor volume defined by ^11^C-methionine (MET) PET versus MRI (20). Perhaps, these findings could explain, to some extent, the distant or out-field failure patterns in patients with gliomas who underwent tumor resection and post-operative radiotherapy. According to the method of target delineation of GBM used by the Radiation Therapy Oncology Group (RTOG), the initial plan tumor volume (PTV) is defined as peri-tumoral edema plus a 2-cm margin, and the dose prescribed to this area is 46 Gy. The boost tumor volume (BTV) is defined as the contrast-enhanced area plus a 2.5-cm margin, and the dose prescribed to this area is 60 Gy [25, 26]. If all of the ^18^F-FLT-positive lesions, including the portions not visible on MRI, are considered tumor tissues, the creation of PTV and BTV without integrating ^18^F-FLT PET would have resulted in an insufficient PTV in two (10.53%) patients and insufficient BTV in five (26.32%) patients. These findings may have significant implications in the treatment strategy for patients with resected malignant glioma, for whom a major obstacle to effective radiation therapy is the inability to delineate the target volume precisely.

Presently, for anatomical delineation of target volumes and identification of organs at risk, MRI provides useful information and is considered the standard tool for the diagnosis and target volume delineation in gliomas. However, evidence has shown that the margin of the contrast-enhancement area or the peri-tumoral edema of the malignant gliomas defined by MRI was not sufficient [27]. Hochberg [28] reported that the malignant cells had the propensity to invade the peri-tumoral edema or normal-appearing brain parenchyma. Several reports have also shown that tumors infiltrate into areas congruent with an abnormal signal on MRI images, a finding confirmed by stereotactic biopsies [29, 30]. The cause may be due to the frequent inability to distinguish a persistent tumor from reparative changes after surgery, and the true extension of the remaining tumor may be under- or overestimated by MRI [31].

PET, as a molecular imaging tool, provides another way to visualize residual tumor in post-operative glioma patients. ^18^F-FDG is the most widely used PET tracer in various malignancies. However, the specificity of ^18^F-FDG is low for brain tumors because of its high uptake in the normal brain cortex. ^11^C-MET, a natural amino acid, is another radiopharmaceutical that can be used for brain tumor imaging [32]. It is taken up avidly by glioma cells, and the uptake by normal brain tissue is relatively low. ^11^C-MET PET has shown promise for delineating the margins of gliomas [33]. It has been suggested that ^11^C-MET PET has greater accuracy in outlining the true extent of viable tumor tissue compared with MRI and CT [34]. Tumors in patients tended to progress in regions that showed high ^11^C-MET uptake but were not covered by the high radiation dose from radiation therapy [35]. However, the short half-life of ^11^C limited the application of this tracer in clinical practice.

The 109-minute half-life of ^18^F makes ^18^F-FLT PET scanning possible at centers without an in-house cyclotron facility and makes this tracer ideal for brain imaging in oncology. There is a sharp border between residual tumor tissue and healthy brain tissue because of the low uptake of ^18^F-FLT in intact brain tissue. Thus, ^18^F-FLT PET is considered an attractive imaging method for malignant brain tumors. Earlier studies have shown that ^18^F-FLT PET has a high sensitivity and specificity for diagnosing high-grade gliomas, and the uptake of ^18^F-FLT was correlated with tumor proliferation [36]. Regarding post-operative radiation therapy planning, it is desirable that the additional information from ^18^F-FLT PET can help correct the evaluation of the surgical results and help define the gross tumor volume (GTV) and clinical tumor volume (CTV) with high accuracy, which is impossible to achieve 1–8 weeks after surgery using anatomical imaging alone [13]. In our study, as mentioned above, the volume of residual tumor defined using ^18^F-FLT PET images was different from that drawn on MR images. The latter finding was consistent with that in the study by Idema *et al*. [37], which observed that the intracerebral uptake of ^18^F-FLT was not limited to areas of contrast enhancement as seen on MRI and, in fact, exceeded the area of contrast enhancement on MRI in most cases. These findings indicated that ^18^F-FLT PET provides additional information compared with MRI in defining target volumes in high-grade glioma, particularly for the boost volumes.

In addition to the above findings, we found that the extension of ^18^F-FLT uptake outside of the MRI abnormal signal areas was not distributed uniformly around the Vol-T1 and Vol-T2 (Figs. 3 and 4). Therefore, a uniform margin for PTV around the target drawn on MR images does not ensure covering the residual tumor adequately in the radiation fields. There is an important role for ^18^F-FLT PET in the delineation of residual disease from the post-surgical changes that, consequently, will influence target volume delineation in radiotherapy. The latter observation may be the most crucial for decreasing recurrence and improving prognosis, including both the survival time and quality of life.

In our study, we found that the integration of ^18^F-FLT- PET into tumor volume delineation for gliomas had another important effect—improved sparing of the normal brain tissue. Contrast-enhancement and edema regions in MRI extended outside the ^18^F-FLT uptake region in 16 patients (84.2%) and 19 patients (100%), respectively. The mean volumes of the MRI abnormal areas beyond the high ^18^F-FLT uptake region were 6.16 cm^3^ on T1-weighted MRI and 21.78 cm^3^ on T2-weighted MRI. If the T1 abnormal enhancement and T2 hyperintensity areas outside the ^18^F-FLT uptake were a result of the surgery, these regions should not be included in the GTV for radiotherapy planning and should be spared from the high radiation dose.

One of the limitations of ^18^F-FLT PET image might be that a disruption of the blood—brain barrier is required for tumor targeting. Especially for low-grade gliomas, this requirement can be a restricting factor [36]. For this reason, only high-grade gliomas were included, in which the BBB is always disrupted. Another limitation of our study was the small number of subjects enrolled. However, our purpose was to evaluate whether ^18^F-FLT PET might be useful for detecting the presence of residual tumor in comparison with MRI. The discordance between the volumes of residual disease defined by ^18^F-FLT PET and MRI provides justification that ^18^F-FLT PET may be able to provide complementary information for treatment planning, and the use of multiple imaging modalities will lead to the most accurate delineation of the target volume. The third potential criticism of our study is that it is unclear whether the ^18^F-FLT-PET activity is correlated with regions at high risk for progression, due to the lack of data on subsequent failures. We will follow up to verify this point. Indeed, if tumor progression is documented in the ^18^F-FLT uptake regions, administering a simultaneous integrated boost to this area may be logical, as shown in mathematical models indicating that dose escalation has a possible positive effect on survival [38, 39]. In addition to this, our study did not perform dynamic PET scanning, although the proliferation index correlates best with the Ki derived from dynamic PET [40]. Considering that previous studies have shown a good correlation between the SUV_max_ and Ki-67 index (15), and that the calculation of the residual tumor volume does not require a kinetic model, use of a single time point for PET scanning was acceptable in our study.

## Conclusions

The post-operative residual tumor volumes defined by ^18^F-FLT uptake in PET images and abnormalities on post-operative MR images were not in complete concordance. ^18^F-FLT PET provides additional information, compared with MRI, to the target volume definition in high-grade gliomas, particularly for the boost volumes. Incorporation of ^18^F-FLT PET data into the target volume definition may potentially improve residual tumor detection, as well as improve tumor control while reducing complications.

## Supporting Information

S1 Table Patient characteristics and results of volumetric measurements of T1 and T2-weighted MRI vs. FLT-PET in 19 patients with resected malignant gliomas. (PDF)
