# Supplementary material for: 18F-Fluorothymidine PET-CT for Resected Malignant Gliomas before Radiotherapy: Tumor Extent according to Proliferative Activity Compared with MRI
Source: PLoS One. 2015 Mar 4;10(3):e0118769. doi: 10.1371/journal.pone.0118769 (PMC4349865; doi:10.1371/journal.pone.0118769)
Supplement: S1 Table — (PDF) [file pone.0118769.s001.pdf]

Table_S1. Patient characteristics and results of volumetric measurements of T1 and T2-weighted MRI vs. FLT-PET in 19 patients with resected malignant gliomas.

Patient No	Gender	Age	WHO grade	Vol-T1	Vol-PET	Vol-T2	Vol-(T1 minus PET)	Intersection Vol-T1/PET	Vol-(PET Minus T1)	Vol-(T2 minus PET)	Intersection Vol-T2/PET	Vol-(PET minus T2)
1	F	38	III	7.5	4.7	25.6	2.8	4.7	0	20.9	4.7	0
2	M	54	IV	2.2	2.2	8.5	0	2.2	0	6.3	2.2	0
3	M	63	IV	20.8	20.8	36.9	0	20.8	0	16.1	20.8	0
4	M	40	III	68.2	15.5	98.7	52.7	15.5	0	83.2	15.5	0
5	F	47	IV	7.8	59.4	106	0	7.8	51.6	57.6	48.4	11
6	M	52	IV	12.4	32.9	28.8	7.2	5.2	27.7	13.4	15.4	17.5
7	M	36	III	3.5	8.8	14.7	1.4	2.1	6.7	6.1	8.6	0
8	M	56	IV	1.9	4.3	9.5	0.9	1	3.3	5.2	4.3	0
9	F	58	IV	1.7	1.8	3.9	1.2	0.5	1.3	2.1	1.8	0
10	M	20	IV	2.9	3.6	3.3	1.1	1.8	1.8	0.8	2.5	1.1
11	M	43	III	4.2	7.8	15.3	1.9	2.3	5.5	7.5	7.8	0
12	M	64	IV	6.4	6.9	9.9	0.6	5.8	1.1	4	5.9	1
13	M	76	IV	14.3	22.9	44.8	2.3	12	10.9	26.8	18	4.9
14	F	49	IV	3.2	4.8	31.4	1.2	2	2.8	26.6	4.8	0
15	M	55	III	22.8	28.5	69.6	8.8	14	14.5	51.3	18.3	10.2
16	F	56	IV	10.48	6.2	5.4	4.28	6.2	0	3.5	1.9	4.3
17	F	51	IV	4.8	3.3	10.4	2.9	1.9	1.4	8.1	2.3	0
18	M	65	III	10.4	9.8	29.8	8	2.4	7.4	20	9.8	0
19	M	50	III	42.9	33.5	72.6	19.8	23.1	10.4	54.3	18.3	15.2
